# Hormone Signaling and Its Interplay With Development and Defense Responses in *Verticillium*-Plant Interactions

**DOI:** 10.3389/fpls.2020.584997

**Published:** 2020-11-04

**Authors:** Nikhilesh Dhar, Jie-Yin Chen, Krishna V. Subbarao, Steven J. Klosterman

**Affiliations:** ^1^Department of Plant Pathology, University of California, Davis, Salinas, CA, United States; ^2^Department of Plant Pathology, Institute of Plant Protection, Chinese Academy of Agricultural Sciences, Beijing, China; ^3^United States Department of Agriculture, Agricultural Research Service, Salinas, CA, United States

**Keywords:** Verticillium wilt, *Verticillium*-host interaction, plant defense response, growth, development, phytohormones, hormone signaling pathways, cross-talk

## Abstract

Soilborne plant pathogenic species in the fungal genus *Verticillium* cause destructive Verticillium wilt disease on economically important crops worldwide. Since *R* gene-mediated resistance is only effective against race 1 of *V. dahliae*, fortification of plant basal resistance along with cultural practices are essential to combat Verticillium wilts. Plant hormones involved in cell signaling impact defense responses and development, an understanding of which may provide useful solutions incorporating aspects of basal defense. In this review, we examine the current knowledge of the interplay between plant hormones, salicylic acid, jasmonic acid, ethylene, brassinosteroids, cytokinin, gibberellic acid, auxin, and nitric oxide, and the defense responses and signaling pathways that contribute to resistance and susceptibility in *Verticillium*-host interactions. Though we make connections where possible to non-model systems, the emphasis is placed on *Arabidopsis*-*V. dahliae* and *V. longisporum* interactions since much of the research on this interplay is focused on these systems. An understanding of hormone signaling in *Verticillium*-host interactions will help to determine the molecular basis of Verticillium wilt progression in the host and potentially provide insight on alternative approaches for disease management.

## Introduction

**Figure 1:**
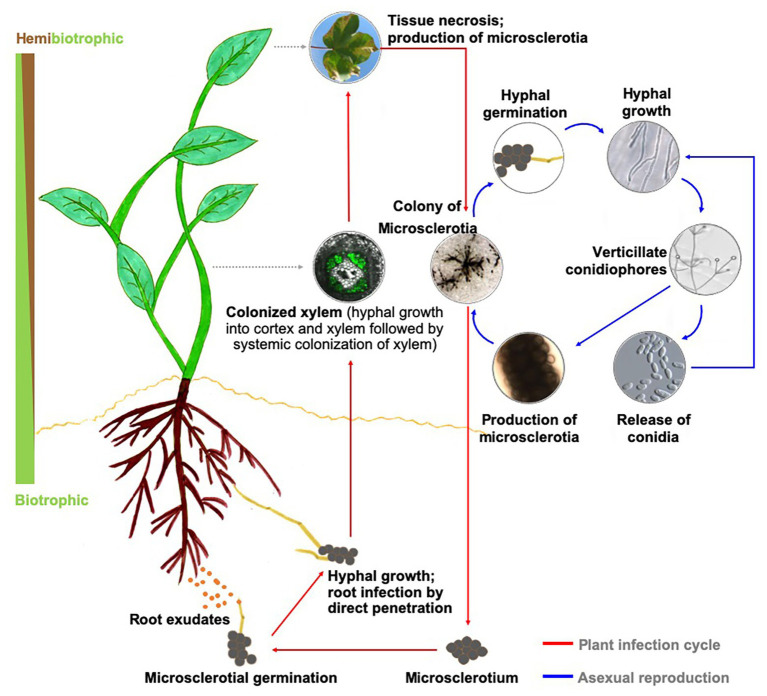
Disease cycle in a generic host and biology of *Verticillium dahliae*. Upon perceiving signals in host root exudates, microsclerotia of *Verticillium* sp. germinate, and the resulting hyphae enter the plant through direct penetration or natural openings and progresses to colonize the xylem. During this biotrophic phase of its life cycle, the pathogen utilizes the host nutrients to propagate and achieves systemic spread in the host xylem. As the rapidly expanding pathogen overtakes the host defenses, the infected plant succumbs to the disease and eventually dies. This period in the infection cycle also overlaps with establishing the necrotrophic phase of *Verticillium* sp., during which the pathogen actively reproduces. As the host nutrient availability status recedes, the fungus forms microsclerotia for long-term survival of the pathogen.

The genus *Verticillium* is composed of 10 species that cause Verticillium wilt on over 200 economically important plant species worldwide (Bhat and Subbarao, 1999; Klosterman et al., 2009; Inderbitzin et al., 2011; Inderbitzin and Subbarao, 2014). Verticillium wilt results in extensive yield or plant losses in many crops, including cotton, potato, tomato, eggplant, lettuce, spinach, alfalfa, strawberry, oilseed rape, canola, sunflower, olive, and also numerous dicotyledonous trees and shrubs of ornamental value (Fradin and Thomma, 2006; Klosterman et al., 2009). *Verticillium dahliae*, the most destructive of the 10 species within the genus *Verticillium* (Inderbitzin et al., 2011; Inderbitzin and Subbarao, 2014), is a soilborne pathogen that produces spores (conidia) during mycelial growth and growth within the xylem, and microsclerotia, the thick-walled, and melanized dark brown to black structures for long-term survival in the soil environment (Short et al., 2015). Seed borne transmission of the microsclerotia has also been demonstrated (Vallad et al., 2005) and the potential pathogen spread *via* infested seed is low in case of lettuce but can occur on a transcontinental scale with infested spinach seed (Atallah et al., 2010; Short et al., 2015). In response to host root exudates, microsclerotia germinate and infect the host roots by direct penetration (Fradin and Thomma, 2006; Klosterman et al., 2009). Once the pathogen reaches the xylem, it systemically spreads within the host (Figure 1).

Resistance to Verticillium wilt is mediated by a dominant gene in several hosts, including tomato, sunflower, potato, and lettuce, among others (Hayes et al., 2011), but the pathogen eventually overcomes this resistance. Based on the response of differential host cultivars, *V. dahliae* has been divided into two races (Vallad et al., 2006; Maruthachalam et al., 2010; Sandoya et al., 2016) and an additional race was described on tomato (Usami et al., 2017). Races 1 and 2 were subsequently correlated with nondefoliating or defoliating phenotypes on cotton (Hu et al., 2015), but this correlation is not perfect as many nondefoliating strains belong to a race different from race 1 (Zhang et al., 2019). Identified resistance genes have been incorporated into tomato resulting in control of race 1 (Diwan et al., 1999; Kawchuk et al., 2001; Fradin et al., 2009). Race 1 resistance in lettuce has been identified and efforts are currently underway to incorporate this resistance into elite cultivars (Hayes et al., 2007, 2011; Sandoya et al., 2016). However, this race-dependent host resistance in tomato was compromised within a few years following its deployment (Grogan et al., 1979). Absence of safe and cost-effective control methods warrant the development of novel disease management techniques. Effectors of *V. dahliae* and their subsequent host defense components in plants have been identified (Fradin et al., 2011; de Jonge et al., 2012) and are important in the fight against Verticillium wilts. Thus, several approaches, including a thorough understanding of the signaling networks and discovery of novel genes that confer quantitative disease resistance against this pathogen are currently being explored.

Plant hormones are a class of chemical compounds similar to animal hormones that are required in minute quantities to regulate growth and development. Plant hormones also play a central role in regulating defense responses against abiotic and biotic stresses, thereby integrating various aspects of plant health. Due to the sessile nature of plants and the central role hormones play in connecting the signaling networks governing an array of processes, precise execution of signal output is required for the healthy plant growth, successful completion of its life cycle culminating with the production of seeds for subsequent generations. External stimuli such as abiotic and biotic factors, including environmental factors, microbes, pests, and herbivores, affect not only the hormone signaling networks but also the cellular hormone levels. In addition to the classical plant hormones such as ethylene (ET), auxin (AUX), cytokinin (CTK), gibberellic acid (GA), and abscisic acid (ABA), research in the past two decades has led to the discovery of new hormones such as salicylic acid (SA), jasmonic acid (JA), brassinosteroids (BR), nitric oxide (NO), strigolactones (STR), karrikins (KAR), and others including peptide hormones and polyamines (Shigenaga and Argueso, 2016; Berens et al., 2017). Interestingly, many of these hormones are produced by plant pathogenic microbes as secondary metabolites and some pathogens may overcome host defense responses with a combination of effectors and interference with host hormone signaling. In contrast, some effectors produced by plant pathogens directly influence hormone levels or the hormone signaling network components to manipulate host resources to their advantage (Shigenaga and Argueso, 2016; Berens et al., 2017). The nature of these interactions determines the outcome of host-pathogen interactions while also affecting the hormone signaling pathways that integrate processes such as nutrient acquisition, defense, growth, and development.

Plant-microbe interactions can be manipulated by the application of plant hormones directly or by interference in hormone signaling processes to assess responses that affect plant productivity as well as defense responses (Delaney et al., 1994; Li et al., 1996; Tjamos et al., 2005; Johansson et al., 2006). A better understanding of plant hormonal responses to *Verticillium* spp. in both susceptible and resistant interactions will aid in effective management of Verticillium wilt while maintaining crop yields. In this review, we examine historical and novel developments on plant hormonal changes in response to pathogenic *Verticillium* species. We present the known host genetic components, defense strategies, and molecular mechanisms underlying defense responses in relation to hormone signaling pathways and identify research areas for the future.

## Hormone Signaling During *Verticillium* Infection

### Salicylic Acid Signaling

SA plays a central role in plant defense responses including systemic acquired resistance (SAR; Delaney et al., 1994; Cao et al., 1997; Vlot et al., 2009). Though SA-mediated pathways regulate defense against biotrophic and hemibiotrophic pathogens, they play ancillary roles against necrotrophic pathogens (Glazebrook, 2005). SA pathway components are required for resistance against *Verticillium* spp. in *Arabidopsis*, tomato, and cotton (Johansson et al., 2006; Fradin et al., 2009; Gao et al., 2011; Dhar et al., 2019). In *Arabidopsis-V. longisporum* interactions, SA levels have been shown to be augmented in an *Arabidopsis* genotype-specific manner following inoculations with the pathogen (Haffner et al., 2014). Similarly, levels of SA and its glucoside (SAG) were elevated in the root xylem sap, hypocotyl, and shoots in *Brassica napus* following *V. longisporum* inoculations, while JA and ABA levels in the xylem sap remained unaffected (Ratzinger et al., 2009). While confirming these results, Zheng et al. (2019) further demonstrated that the introduction of bacterial *NahG* (encoding salicylate hydroxylase that converts SA to catechol) into the host plant depleted SA and increased susceptibility to *V. longisporum*. Thus, a threshold SA level may be required to confer resistance against this pathogen.

The phenylpropanoid pathway is the major contributor to SA biosynthesis in potato. The expression of *phenylalanine ammonia-lyase 1* (*PAL1*) and *phenylalanine ammonia-lyase 2* (*PAL2*) is upregulated in potato following *V. dahliae* infection. The response of these genes, along with other SA signaling genes such as *pathogenesis related* genes including *PR1*, *PR2*, and *PR5* are induced strongly in the moderately resistant cultivars compared to the susceptible cultivars (Derksen et al., 2013). Furthermore, on cotton calli, exogenous SA application led to the protection of callus cells against the deleterious effects of *V. dahliae* (VD) toxin (Zhen and Li, 2004). A genome-wide association study (GWAS) in cotton determined that the cotton *glutathione S-transferase 9* (*GaGSTF9*) was a positive regulator of defense against Verticillium wilt, following enhanced SA signaling in response to the pathogen (Gong et al., 2017). Not only are GSTs inducible by exogenous SA application, but also the *OxGSTF9* plants showed higher induction of SA levels *in planta* upon challenge with *V. dahliae*, indicating that GSTs play a significant role in SA-mediated defense in *V. dahliae*-cotton interactions.

The plant *nonrace specific disease resistance 1* (*NDR1*) gene is central to SA-mediated defense responses and is required for resistance against plant pathogenic bacteria, fungi, and nematodes (Century et al., 1995; Gao et al., 2011; Dhar et al., 2019). *NDR1* is required for a reactive oxygen species (ROS)-dependent hypersensitive response (HR) and the accumulation of SA in response to pathogens (Century et al., 1995). Both *NDR1* and *enhanced disease susceptibility 1* (*EDS1*) signaling pathways are required for the signaling cascade downstream of the *Verticillium race1 resistance* (*Ve1*)-mediated defense responses in tomato, even though these SA signaling components mediate defense responses to two different classes of nucleotide-binding leucine-rich repeat (NBS-LRR) type defense proteins in plants (Aarts et al., 1998; Hu et al., 2005; Fradin et al., 2009). *GbEDS1*, the ortholog of *A. thaliana EDS1* in cotton, is induced by both *Verticillium* infection and SA. While ectopic expression of *GbEDS1* led to increased resistance with increased levels of SA and the ROS hydrogen peroxide, silencing of the gene produced the opposite effect (Yan et al., 2016). Following *V. dahliae* infection, the levels of *AtEDS1*, *AtNDR1*, and *BRI1-associated receptor kinase 1* (*AtBAK1*) were significantly upregulated in transgenic *Arabidopsis* plants ectopically expressing the cotton *GbEDS1*, while *GbEDS1*-silenced cotton plants showed the opposite trend. Although both *EDS1* and *NDR1* are known to regulate different classes of *R* gene-mediated resistance pathways, both of these genetic components are required for resistance against *V. dahliae* in an SA-associated manner (Aarts et al., 1998; Fradin et al., 2009; Yan et al., 2016).

Furthermore, a mutation in the allotetraploid cotton *5-aminolevulinic acid dehydratase* (*ALAD*) gene, encoding an enzyme that localizes into the chloroplast and functions at an early step in chlorophyll and heme biosynthesis, resulted in high levels of ROS, SA, and constitutive expression of PRs leading to increased resistance against *V. dahliae*. High levels of ROS *via* auto oxidative decomposition in a synergistic amplification loop, aided by upregulation of positive regulators of SA biosynthetic genes, including *EDS1*, *phytoalexin deficient 4* (*PAD4*), and *PAL* were proposed to underlie the increased tolerance to *V. dahliae* (Chai et al., 2017).

Further evidence for SA-mediated defense response against *Verticillium* infection was described by Liu et al. (2014), who showed that *Arabidopsis* plants expressing the *Pseudomonas putida NahG* resulted in increased susceptibility of the plants. The authors inferred that suppressing plant SA-mediated defense responses downstream of SA biosynthesis may be the cause of this increased susceptibility. In the *V. longisporum*-infected oilseed-rape *Brassica napus* system as well, the SA-mediated defense responses were required for resistance (Zheng et al., 2019). While increased levels of SA were observed in the resistant cultivar compared with the susceptible cultivar in early asymptomatic stages, this difference was nullified at later stages of infection. As with cotton-*Verticillium* interactions, *V. longisporum*-treated transgenic *B. napus* plants expressing the SA catabolic *NahG* resulted in increased susceptibility to the pathogen (Zheng et al., 2019), reiterating the importance of SA-mediated responses in host resistance against *Verticillium*.

In yet another study involving *Arabidopsis*, mutations in genes for SA biosynthesis and accumulation did not result in a significant loss of resistance to *V. dahliae*; however, a mutation in the SA receptor *nonexpresser of PR genes 1* (*NPR1*) resulted in the destabilization of this protein leading to susceptibility (Johansson et al., 2006), indicating that SA perception is equally important. Though *Arabidopsis* plants with a mutation in *NPR1* increased susceptibility to *Verticillium* (Johansson et al., 2006), cotton plants with ectopic expression of *AtNPR1* heightened resistance to only the non-defoliating strains of *V. dahliae*; with resistance to the defoliating strain remaining unaltered (Parkhi et al., 2010). Similarly, ectopic expression of *NPR1* from wild eggplant *Solanum torvum* (*StoNPR1*) in a susceptible potato cultivar resulted in increased resistance to *Verticillium*, whereas transgenic *StoNPR1*-RNAi expressing potato plants showed increased susceptibility (Deng-Wei et al., 2014). Thus, fortification of resistance to *Verticillium* is likely dependent on the levels of *NPR1* as inferred from studies on multiple host species. Root colonization by the plant-growth-promoting rhizobacterium (PGPR) *Paenibacillus alvei* strain K165 activates induced systemic resistance (ISR) in a SA-dependent manner to increase resistance in *Arabidopsis*. The ISR activity of K165 was dependent on SA pathway biosynthesis genes *salicylic acid induction deficient 1* (*SID1*), *salicylic acid induction deficient 2* (*SID2*), and the receptor gene *NPR1*, since a loss-of-function of these genes abrogated the effect in K165-treated mutant plants (Tjamos et al., 2005).

While multiple genetic components, biochemical pathways, metabolites, and the SA signaling components play important roles in modulating defense responses against *Verticillium* spp., transcriptional reprograming after infection is also associated with SA signaling. Changes in plant transcription and translation machinery by pathogen infection are routinely correlated with alterations of phytohormone levels. A 60S ribosomal subunit protein (RPL18) necessary for intracellular protein biosynthesis was shown to regulate resistance against *Verticillium* in both cotton and *Arabidopsis* in a SA-dependent manner (Gong et al., 2017). While the cotton *GaRPL18* was strongly and rapidly induced by the application of SA, levels of SA were significantly lower in the plants where *GaRPL18* was silenced. Additionally, a strong induction of the SA-related genes but not the JA or ET pathway genes was found in *V. dahliae*-challenged *Arabidopsis* plants over-expressing *GaRPL18*. In contrast, pretreatment of such plants with SA resulted in increased resistance to the pathogen in these transgenic plants relative to the treated wild type plants (Gong et al., 2017).

Many pathogens employ effector molecules that alter the hormone levels in plants as an infection strategy (Shigenaga and Argueso, 2016). Consequently, a search for effector proteins that might be employed by *Verticillium* spp. to suppress SA levels in the host led to the discovery of a *V. dahliae isochorismate synthase 1* (*VdIsc1*) that was upregulated during early infection stages (Liu et al., 2014). When cotton plants were inoculated with a *VdIsc1* deletion mutant, the levels of the host SA and SAG levels, as well as that of the SA marker, *PR1*, were significantly upregulated. Another secretory protein *V. dahliae* secretory protein 41 (*Vd*SCP41) targets the master immune regulators *cam-binding protein 60-like G* (*CBP60g*) and *SAR deficiency 1* (*SARD1*) that regulate the SA-mediated defense response sector in multiple plant species, including *Arabidopsis* and cotton (Qin et al., 2018). While *CBP60g* and *SARD1* bind directly to the promoters of *SID2*, *enhanced disease susceptibility 5* (*EDS5*), and *NPR1*, the intracellular fungal effector *Vd*SCP41 binds to these upstream transcription factors (TFs; *CBP60g* and *SARD1*) and regulates their activity inside the host cell (Qin et al., 2018). Thus, SA signaling plays a significant role during plant-*Verticillium* interactions. SA perception through *NPR1* and pathogen-mediated alterations in host SA levels seem to determine the outcome of resistance against this pathogen.

### Role of Jasmonic Acid Signaling

Jasmonic acid and the related jasmonates are a group of plant hormones that play a role in plant response to biotic stress, abiotic stresses, and plant developmental processes and share structural similarity to the prostaglandin class of metazoan hormones in animals (Chini et al., 2007). JA, which is a major constituent of the flower scent of jasmine, is synthesized in chloroplasts from membrane lipids in plants through the octadecanoid pathway and is perceived by *coronatine-insensitive 1* (*COI1*; Berens et al., 2017). JA plays a major role in defense against the necrotrophic pathogens, herbivores, insect pests and wounding, as well as in plant reproduction, including pollen fertility (Xie et al., 1998; Berens et al., 2017).

Pretreatment of cotton plants with methyl-jasmonate (MeJA) before infection with *V. dahliae* confers resistance. This resistance was attributed to the activation of basal resistance mechanisms in the host (Li et al., 1996). Subsequent work established a role for JA and ET signaling pathway components in resistance against *V. longisporum* in *Arabidopsis* (Johansson et al., 2006), while brassinosteroid (BR) and JA regulate resistance against this pathogen in cotton (Gao et al., 2013a). JA biosynthetic genes, including *lipoxygenase 3* (*LOX3*), *lipoxygenase 4* (*LOX4*), and *oxophytodienoate-reductase 3* (*OPR3*), were induced within 24 h post-treatment with *V. dahliae*. However, differences in the levels of active JA and jasmonoyl isoleucine (JA-Ile) were detected only 3 weeks post-inoculation, indicating that the upregulation of the genes involved in pathogen mediated phytohormone levels are induced early in the infection when the fungus was in the pre-vascular growth phase inside the host root tissue (Scholz et al., 2018). JA pathway mutants like *jasmonate resistant 1* (*jar1*), *coronatine insensitive 1* (*coi1*), and *cytochrome P450*_*family 94_subfamily B_polypeptide 3* (*cyp94B3*) exhibited reduced colonization and lower fungal biomass in roots and thus were resistant to *V. dahliae* (Scholz et al., 2018). Based on these results, the authors hypothesized that the pathogen might require JA components to promote necrosis type cell death in the later phases of infection, while an absence of JA might lead to activation of SA-mediated responses required for host resistance by prolonging the biotrophic phase of *V. dahliae*.

A nuclear localizing TIR-NBS-LRR protein from a cotton resistant cultivar with homology to *Arabidopsis dominant suppressor of camta3 1* (*DSC1*) and tobacco *NgN* regulates resistance to *V. dahliae* in both *Arabidopsis* and cotton. While *GhDSC1* was specifically upregulated by *V. dahliae* in the resistant cultivar, it was strongly induced by the application of JA but not to the same extent by any other hormone tested. While the *Arabidopsis dsc1* mutant was susceptible to *V. dahliae*, ectopic expression of *GhDSC1* led to increased resistance, and *GhDSC1* complemented the susceptible phenotype of the *Arabidopsis dsc1* mutant. *DSC1* mediated resistance to *V. dahliae* involved the accumulation of ROS. Studies using transgenic *GhDSC1* ectopic expression showed that it complemented the host defense response to the pathogen, which was activated through the JA signaling pathway. Furthermore, the activation of *DSC1* was negatively regulated by *calmodulin-binding transcription activator 3* (*GhCAMTA3*), which shares similar activation kinetics and regulatory stimuli with those of *DSC1*, leading the authors to hypothesize that the function of these two defense regulators may be coupled through modulation of JA signaling pathway in cotton (Li et al., 2019).

Many components of plant metabolic pathways contribute to defense through regulating the hormone-mediated responses. Lignin biosynthesis, a branch of the phenylpropanoid metabolism, reinforces plant cell walls, which serve as a natural barrier against pathogen penetration, and the secondary metabolic products associated with lignin production are upregulated in *Verticillium*-tomato interactions (Hu et al., 2018a, 2019). The cotton *LACCASE 1* (*GhLac1*) functions as a lignin polymerization enzyme and was strongly induced by *V. dahliae* and JA, but not SA, in the roots of the cotton plants (Hu et al., 2018a, 2019). Loss-of function in this genetic component in cotton led to a redirection of the metabolic flux resulting in the accumulation of JA and JA-Ile that was further enhanced during infection with *V. dahliae*. This increased resistance against *V. dahliae* and cotton bollworm (Hu et al., 2018a). Another genetic component that affects the lignin content and JA signaling is the cotton *umecyanin-like 1* (*GhUMC1*) encoding a blue copper-binding protein. While this gene is highly expressed in the root tissues, infection by *V. dahliae* leads to the downregulation of the gene. Moreover, the JA application upregulated this gene, but the silencing of this gene led to reduced expression levels of genes involved in JA biosynthesis and signaling in general (*LOX*s, *MCY2*, and *OPR3*). The *GhUMC1* silenced plants were compromised in their ability to induce oxidative burst and synthesize lignin in response to *V. dahliae*, resulting in enhanced susceptibility (Zhu et al., 2018).

Some plant genes that have more indirect roles in SA and JA regulation act as regulators of defense responses against *Verticillium* infection. The peroxisome-localized cotton *calcium-dependent protein kinase 33* (*GhCPK33*) was specifically induced by *V. dahliae* strain V991 and served as a negative regulator of resistance. Induction of this gene resulted in the destabilization of the JA biosynthesis gene *GhOPR3*, and impaired the JA dependent defenses and rendered the host susceptible (Hu et al., 2018b). Another cotton *cytochrome P450 CYP82D* family gene, *silence-induced stem necrosis* (*GhSSN*), was induced by JA and *V. dahliae* in the roots of susceptible cotton but was downregulated in the resistant line. Furthermore, silencing of this gene induced HR-like lesion mimic mutants that induced hyperaccumulation of JA, which in turn led to the suppression of the SA signaling and enhanced resistance against *V. dahliae*, suggesting that JA rather than SA plays a critical role in resistance against *V. dahliae* (Sun et al., 2014). The cotton stearoyl acyl-carrier-protein desaturase *suppressor of SA insensitive 2* (*GbSSI2*) was strongly induced in the resistant *G. barbadense* cv7124 following *V. dahliae* infection. While the silencing of the *GbSSI2* led to increased susceptibility to *Verticillium* accompanied by downregulation of the JA biosynthesis and signaling components, there was a concomitant increase in the endogenous SA levels (Gao et al., 2013a). The above results indicate that *V. dahliae* infection activates the JA signaling pathway in cotton, and a loss of *GbSSI2* function led to the suppression of defense responses and the loss of resistance (Gao et al., 2013b).

The mediator complex is a conserved multiprotein cofactor of RNA polymerase II and regulates transcription. It consists of 20–30 subunits that form four mediator subcomplexes, which in turn is subjected to dynamic regulation intertwined with hormone signaling. Mediator subunits influence multiple plant processes like development, flowering, non-coding RNA processing, secondary metabolism, and defense response to various abiotic and biotic stresses (An and Mou, 2013; Li et al., 2018b). The expression of one such cotton *cyclin-dependent kinase E* (*GhCDKE*), a subunit of the cotton mediator complex, is induced by *V. dahliae* infection and JA treatment. Not only were JA-mediated responses compromised in the *CDKE*-silenced cotton lines, but also the *GhCDKE* over-expressing *Arabidopsis* plants were more sensitive to JA, indicating that *GhCDKE* functions in the JA signaling pathway. The silencing of *GhCDKE* in cotton led to enhanced susceptibility to *V. dahliae*, while its ectopic expression in *Arabidopsis* led to enhanced resistance against the pathogen in a JA-dependent manner (Li et al., 2018b).

Transcriptome profiling of cotton plants infected with *V. dahliae* identified *homeodomain transcription factor 1* (*HDTF1*), a negative regulator of JA signaling that is preferentially expressed in the leaf tissues. This gene is downregulated by *V. dahliae* infection and MeJA treatment while it is induced by SA treatment. The silencing of *HDTF1* increased the expression of genes involved in JA biosynthesis and accumulation, leading to activation of JA-mediated signaling, though it was not accompanied by an alteration in SA accumulation or SA signaling. Thus, increased resistance of cotton to *V. dahliae* and *B. cinerea* in the *HDTF1*-silenced plants was attributed to enhanced JA signaling (Gao et al., 2016).

Cotton *homeobox 12* (*GhHB12*) encodes a plant-specific homeodomain-leucine zipper (HD-ZIP) family protein that affects various plant developmental processes and is induced by *V. dahliae* infection and MeJA application. *GhHB12* over-expressing plants displayed increased susceptibility to *V. dahliae*, which correlated with the stronger suppression of the JA-responsive, defense-related genes *jasmonate-zim-domain protein 2* (*GhJAZ2*) and *pathogenesis-related 3* (*GhPR3*), as well as decreased lignin content in the stem of infected cotton plants (He et al., 2018a).

Another transcription factor that represses JA-mediated defense response in response to *V. dahliae* infection of cotton is the plant-specific NAC transcription family member *ATAF1*. Though *GhATAF1* expression increased upon treatment with SA and JA in conjunction with *V. dahliae*, over-expression of *ATAF1* in cotton increased wilting and foliar discoloration indicative of increased susceptibility to the pathogen. Suppression of the JA signal components (including *GhLOX1*, *GhMYC2*, *GhPR3*, and *GhPR4*) by *V. dahliae* infection was accompanied by a concomitant increase in the SA pathway genes (including *GhNDR1*, *GhNPR1*, *GhPR1*, and *GhPR5*), highlighting a branch of defense signaling that involves reciprocal regulation of JA-SA mediated defense responses during *Verticillium* infection (He et al., 2016). The application of JA not only enhanced resistance against *Verticillium* but also inversely affected the growth of cotton plants (Li et al., 2014). Thus, hormonal signaling during pathogen infection could lead to transcriptional reprograming during defense responses to *Verticillium* in cotton and *Arabidopsis*.

Though both SA and JA signaling play a role in defense against *Verticillium* sp., the SA-mediated response appears to take a predominant role during the initial biotrophic phase of infection. During the necrotrophic phase, however, JA-mediated defenses play an active role, indicating that the host defense mechanism has evolved as a layered temporospatial response to counter or restrict the damages from the systemic pathogen spread within the host, followed by a strategic switch in its lifestyle during the subsequent progression of Verticillium wilt.

### Role of Ethylene Signaling

The gaseous plant hormone ET plays a central role in senescence and defense responses to necrotrophic pathogens (Broekaert et al., 2006). Hence, the Verticillium wilt-associated chlorosis/yellowing of the foliage from the loss of chlorophyll prompted investigations into the role of ET signaling during *Verticillium* infection. Most studies on the role of ET in Verticillium wilt involve tomato, cotton, and *Arabidopsis*. Elevated ET levels were observed in tomato plants coinciding with the onset of symptoms (Cronshaw and Pegg, 1976). The production of ET was especially enhanced in the susceptible tomato lines by the culture filtrates fraction that were enriched in protein and pectolytic enzymes (Pegg and Cronshaw, 1976). While ET production was stimulated by only the protein/enzyme fraction of culture eluates, increased wilting and necrosis was observed with other eluted fractions, i.e., containing pectolytic enzyme activity or inactive polysaccharide in plants pretreated with ET. Taken together, these results led to the proposition that ET may act as a toxin synergist in Verticillium wilt of tomato (Pegg, 1976; Pegg and Cronshaw, 1976).

Studies in potato confirmed the role of ET in *Verticillium* pathology. Exposure of potato plants to a low-molecular-mass *Verticillium* toxin from the culture filtrate of *V. albo-atrum* (=*V. nonalfalfae*) revealed correlation of susceptibility to *Vd* toxin with the differential production of ET. Susceptible cultivars produced more ET than tolerant potato cultivars. Inhibiting host ET signaling (perception/biosynthesis) by pharmacological treatments abrogated the toxin-induced and ET-mediated symptom development in the host, proving the involvement of ET in Verticillium wilt (Mansoori and Smith, 2005). Additionally, a necrosis- and ET-inducing protein *V. dahliae* necrosis-and ethylene-inducing protein (VdNEP) protein from a cotton isolate of *V. dahliae* activated both SA- and ET-JA-dependent defense pathways in *Arabidopsis* while strongly inducing the expression of the host *1-aminocyclopropane-1-carboxylic acid synthase 6* (*ACS6*) gene encoding the ET biosynthetic enzyme 1-aminocyclopropane-1-carboxylate synthase (Wang et al., 2004).

Genome-wide analysis of the pathogen later revealed the existence of the necrosis- and ET-inducing-like protein (NLP) family of conserved fungal effectors, of which eight were predicted in *V. dahliae* (Klosterman et al., 2011; Santhanam et al., 2013). Two members of this family (NLP1 and NLP2) expressed inside the host following infection exhibit cytotoxic activity. Furthermore, loss of function of these genes led to defects in conidiophore and aerial hyphae in the pathogen (Santhanam et al., 2013). Additionally, two members of a VdNLP family (VdNLP1 and VdNLP2) of cytolytic toxins induce ROS and promote necrosis in cotton with the induction of SA/JA defense pathways as well as upregulation of ACS6 during *V. dahliae* infection of cotton (Zhou et al., 2012).

Components of the ET signaling pathway-ethylene insensitive 2 (EIN2), ethylene insensitive 4 (EIN4), and ethylene insensitive 6 (EIN6) in *Arabidopsis* positively regulate defense responses against *V. longisporum* (Johansson et al., 2006). This study also shed light on the fact that ET production followed pathogen infection and was independent of ET sensitivity of the host root system, though a higher resistance to the pathogen was observed in the ethylene resistant 1-1 (etr1-1) mutant. Although ET pretreatment did not compromise fungal growth on media, treatment of the pathogen-challenged host with the ethylene biosynthetic precursor 1-aminocyclopropane-1-carboxylic acid (ACC) resulted in a substantial increase in the fresh weight of the inoculated plants. Evidence for the involvement of ET signaling components in Verticillium wilt came from studies involving Never Ripe (SlNr) and ethylene resistant 4 (SlETR4) in tomato, wherein silencing of these ET receptor genes reduced disease incidence, severity, and reduced fungal biomass indicative of increased resistance to *V. dahliae* (Pantelides et al., 2010a). Impaired perception of ET in the *Arabidopsis* etr1-1 mutant led to a significant reduction in pathogen growth and increased resistance to *V. dahliae* (Pantelides et al., 2010b).

In cotton, an ethylene response factor-like gene (GbERF1-like) is induced by *V. dahliae* along with the hormones MeJA and ET, respectively. The GbERF1-like protein acts as a positive regulator of defense against the pathogen by fortifying lignin synthesis upon pathogen challenge (Guo et al., 2016). A cotton major latex protein 28 (GhMPL28) interacted with the cotton ethylene response factor 6 (GhERF6) transcription factor facilitating its binding to the GCC-box element in the promoters of the defense-related genes and positively regulating defense against *V. dahliae* (Yang et al., 2015).

The role of ET during *Verticillium* infection has been contradictory, with some studies indicating a dual role for ET in resistance as well as the promotion of wilt. Studies involving different stages of infection and the timing of ET biosynthesis/perception have elucidated the exact role of this hormone during Verticillium wilt symptom in tomato. Though symptom development and ET production rapidly increased in the first few days after *V. dahliae* inoculation of tomato, these declined thereafter (Robison et al., 2001a). A reduction in the pathogen-induced ET production in tomato plants treated with *V. dahliae* was achieved upon the application of the ET inhibitor aminoethoxy vinyl glycine (AVG) either before or at the time of inoculation. This led to greater resistance, reduced symptom severity, reduced disease, and greater fresh weight of the infected host (Robison et al., 2001a). Complimentary studies have shown that plants transformed with the bacterial *ACC deaminase* gene under root-specific or infection-induced promoters that inhibit ET synthesis, significantly reduced or delayed symptoms when challenged with *V. dahliae*. Tissue or defense-specific induction of this catabolic enzyme degraded the ET precursor ACC, resulting in reduced chlorosis and wilting, increased fresh weight, and overall larger plants (Robison et al., 2001b).

Another study examining the role of *ACS* genes found that the *1-aminocyclopropane-1-carboxylic acid synthase 2* (*ACS2*) and *1-aminocyclopropane-1-carboxylic acid synthase 6* (*ACS6*) genes were upregulated by 2 weeks post-*V. dahliae* infection in *A. thaliana*. The authors further demonstrated a lower endophytic level of *V. dahliae* in mutant plants since mutant plants were more resistant than the wild type plants in an ethylene dependent manner (Poulaki et al., 2020). While the production of ET by the host plant during early stages of *V. dahliae* infection results in the induction of defense responses, ET production post-infection seems to favor enhanced infection. More significantly, the results from this study demonstrate that components of ET biosynthesis, as well as the ET precursor ACC, play an important role during the progression of Verticillium wilts.

Though ET plays a significant role in the plant responses to *Verticillium* spp., the effect of ET signaling that determines the outcome of this interaction seems to depend on the timing, amplitude, and spatial nature of the perception, and relay of the signal during fungal pathogenesis. Perception of the pathogen seems to activate ET signaling along with the induction of ET biosynthesis by multiple *Verticillium* proteins and toxins, which in turn activates the defense responses and induce cell death to limit the pathogen at the site of infection during the early stages of infection. But in later stages of infection, during colonization and the switch to the necrotrophic phase, these responses might, in fact, aid in the establishment of the pathogen.

### Role of Brassinosteroid Signaling

Brassinosteroids are group steroid hormones that are found in both plants and animals. In plants, BR is primarily involved in plant growth, cell differentiation, and developmental processes, including reproduction and senescence, but are most notable for their involvement in the photomorphogenesis of plants. While the receptors for plants are present on the cell surface, the receptors for the animal counterparts are transcription factors that belong to the nuclear receptor family (Wang et al., 2012). However, their role in defense responses to various pathogens has attracted further attention.

Long-term exposure to epibrassinolide reduced or eliminated symptoms of *Verticillium* infected tomato plants while short-term exposure had no effect (Krishna, 2003; Roos et al., 2014). Analyses of cotton calli exposed to *Verticillium* suggests the involvement of BR in ameliorating *Verticillium* toxin-induced stress (Bibi et al., 2017) while BR signaling component *BRI1-associated receptor kinase* (*BAK1*) was shown to be a positive regulator of race-specific disease resistance against the pathogen in tomato (Fradin et al., 2009; Roos et al., 2014). Pretreatment of cotton calli rather than co-/post-treatment with epibrassinolide significantly negated the effects of *Vd* toxin in a pathogen-free model system with a significant increase in photosynthetic pigments and accompanying secondary metabolism (Bibi et al., 2017). A decrease in water and nutrient uptake caused by the clogging of the xylem by *Verticillium* cell wall degrading enzymes is known to induce osmotic stress during *Verticillium* pathogenesis (Bibi et al., 2014). A decline in photosynthesis caused by reduction in leaf surface area as well as the wilting of leaves causes a decline in the overall photosynthetic ability of the plant during *Verticillium* infection contributing to the loss of carbohydrate metabolites that could mitigate the effect of osmotic stress caused by the pathogen in the vascular tissues (Bowden et al., 1990; Hampton et al., 1990; Sadras et al., 2000). Plants try to counter this with an increase in the enzymes involved in carbohydrate metabolism, including sucrose phosphate synthase (SPS), vacuolar/cell wall-bound acid invertase (AI), and cytosolic sucrose synthase (SuSy; Bibi et al., 2014). While *Vd* toxin resulted in a slight increase in these enzymes, the application of epibrassinolide significantly elevated the level of these enzymes. Such induction was achieved by a relatively lower concentration of the hormone applied to the roots, while a higher concentration was required for foliar induction (Bibi et al., 2014). The effect of such osmotic stress is mitigated by the host in part by an increase in production of proline, glycine betaine, and soluble sugar (Goicoechea et al., 2000; Bibi et al., 2014). Further, the application of BR mitigated the growth inhibitory effect of a *Vd* toxin by reducing the damage to chlorophyll and enhancing the rate of photosynthesis as well as transpiration, resulting in increased root and shoot biomass (Bibi et al., 2014).

Disease resistance in cotton may in part depend on brassinolide signaling. *BAK1*, which encodes an LRR-RLK that is associated with the BR receptor *brassinosteroid insensitive 1* (*BRI1*) and belongs to a sub-family of receptor-like kinases (RLKs) supports this hypothesis. Loss-of-function of this gene not only led to cell death accompanied by the production of ROS but also resulted in resistance against the Verticillium wilt in *Arabidopsis*, tomato, and cotton showing that this signaling pathway is conserved across species (Fradin et al., 2009; Gao et al., 2013a; Roos et al., 2014). Yet another study utilizing proteomics analysis led to the discovery of crosstalk between the JA-BR pathways (Gao et al., 2013b). While this work highlighted that BR signaling was activated in the cotton-treated with *Verticillium*, it also showed that the disease resistance in cotton was fortified upon the application of brassinolide while activating the JA signaling pathway, indicating a positive correlation between these two hormone signaling pathways (Gao et al., 2013b).

Among the RLKs that are associated with BR signaling in plants is the *suppressor of BIR1* (*SOBIR1*), which, as the name suggests, was discovered in a genetic screen on *BAK1-interacting receptor-like-kinase 1* (*BIR1*). SOBIR1 is required for resistance against various pathogens and interacts with many receptor-like proteins (RLPs) as well as with *BAK1* to influence cell death and defense response independent of *BIR1* (Gao et al., 2009; Liu et al., 2016). While the cotton homolog of *SOBIR1* gene (*GbSOBIR1*) is induced by *V. dahliae*, a reduction in the levels of expression of this BR pathway gene compromises the host resistance, and ectopic expression results in increased resistance to *V. dahliae* (Zhou et al., 2019). Although the expression of *GbSOBIR1* was enhanced by the SA application, treatment with JA caused its downregulation. A previous study had found a positive effect between BR-induced resistance and activation of JA response upon challenge with the pathogen. Protein-protein interaction studies led to the discovery of a basic helix-loop-helix (bHLH) family transcriptional activator *bHLH171*, and further experiments showed that this protein is phosphorylated by GbSOBIR1 most likely at a serine residue that is required for its transcriptional activity (He et al., 2018b). The homolog of *Arabidopsis SOBIR1* in tomato (*SlSOBIR1*) is required for resistance to *V. dahliae* in a *Ve1*-dependent manner and interacted with a broad range of RLPs. Further, the silencing of this gene resulted in reduced *Ve1* levels leading to impaired HR during *Ve1-Ave* interaction in the plant (Liebrand et al., 2013). Since both *Ve1* and *BAK1* are conserved across plant species and form receptor complexes while contributing to resistance against *V. dahliae*, these two receptor-like proteins may partner with each other in some capacity during host defense responses to Verticillium wilt (Gao et al., 2013b).

Two *Verticillium* glycoside hydrolase 12 (GH12) proteins *V. dahliae* endoglucanase 1 (VdEG1) and *V. dahliae* endoglucanase 3 (VdEG3) act as pathogen-associated molecular patterns and induce immunity-related cell death in tobacco. In conjunction with another *Verticillium* effector carbohydrate-binding module family 1 (CBM1) protein, these GH12 *Verticillium* proteins together manipulate host defense responses involving the BR signaling components BAK1 and SOBIR1. Further, VdEG1 was perceived through the LRR-RLP/SOBIR1/BAK1 complex while VdEG3 was perceived through the LRR-RLKs/BAK1 complex independently of their enzymatic activity, leading to activation of defense response and host cell death. Both were in turn suppressed by the pathogen effector VdCBM1 in a dose-dependent manner to promote infection in the host (Gui et al., 2017). Another *Verticillium* cutinase family protein, *V. dahliae* CUTINASE 11 (VdCUT11) is notably upregulated and functions as a major virulence factor during *V. dahliae* infection in both cotton and tobacco, resulting in significant loss of plant biomass. However, its recognition leading to activation of host defense response leading to host cell death and damage-associated molecular pattern-triggered immunity, in turn, is suppressed by the VdCBM1 effector in a BAK1- and SOBIR1-dependent manner in tobacco plants. This sequence of events thus aids in the establishment of Verticillium wilt in the susceptible host (Gui et al., 2018).

A *Rab GTPase-activating protein 22* (*RabGap22*) from *Arabidopsis* was induced by *V. longisporum* and is required for defense against the pathogen. Further inquiry on the *RabGap22* co-expressed genes revealed the role of somatic embryogenesis receptor-like kinase 3 (SERK3)/BAK1 as a positive regulator of defense responses. Mutation in *BAK1* not only increased the susceptibility but also attenuated the *RabGap22* expression in *V. longisporum*-challenged *Arabidopsis* plants (Roos et al., 2014). The susceptibility of the *rabgap22-1* mutant to this pathogen could be rescued with an application of 24-epibrassinolide, further indicating the importance of this phytohormone signaling pathway in host defense response during *Verticillium* infections.

### Role of Cytokinin Signaling

Since reduced CK levels are associated with yellowing of leaves resulting from loss of chlorophyll, which is a hallmark of senescence and progression of Verticillium wilt, many studies have focused on the correlation of this hormone with senescence and wilt in susceptible hosts. Early studies on cotton plants (*Gossypium hirsutum*) treated with a pathogenic strain of *Verticillium* spp. showed a significant decrease in the levels of CK in the tracheal fluid and in the extract from the aerial tissues including stem and leaves, following leaf symptom development (Misaghi et al., 1972). Consistently lower levels of CK also occur in tomato plants following the onset of Verticillium wilt symptoms (Patrick et al., 1977). Based on these results, it was proposed that a decrease in water potential in the root leads to a reduction in CK levels, which in turn underlies the visible yellowing of the leaves due to chlorosis and loss of pigments during *Verticillium* infection (Patrick et al., 1977).

*Verticillium*-induced premature senescence is accompanied by a decrease in the host CK levels and this occurs because of upregulation of CK-degrading enzymes (Reusche et al., 2013). The expression maxima of these CK degrading enzymes cytokinin oxidase (CKX1, CKX2, and CKX3) correlated with the pathogen-induced decrease in the plant CK pool, trans-zeatin [tZ], in particular, which is the most prevalent CK in the model plant *Arabidopsis*. Reduced CK level was thus predicted to be beneficial for a necrotrophic pathogen or during the necrotrophic phase in the life cycle of a pathogen like *V. longisporum*, which might be a strategy for efficient colonization of the leaves by active induction of senescence (Reusche et al., 2013). External application of a synthetic CK as well as inhibition of the CK degrading CKX activity led to reduced symptoms and proliferation of the fungus on the host (Reusche et al., 2013). Thus, active CK levels seem to play an essential role in mediating defense responses during *Verticillium* infection.

### Role of Gibberellic Acid Signaling

Hyper elongation of the rice stem in response to *Fusarium fujikori* is a striking example of the manipulation of GA signaling by a pathogen (Kurosawa, 1926). The pathogen-derived (plant growth-promoting) gibberellin has been attributed as the cause of bakanae disease on rice (Studt et al., 2013). As another example of the manipulation of GA levels by a pathogen, the interaction of the viral RVD outer capsid protein P2 with a rice gibberellin biosynthesis gene *ent-kaurene oxidase* (*KAO*) led to a reduction in the levels of this hormone during infection of the rice dwarf virus (Zhu et al., 2005).

Challenge by a pathogen results in a “growth versus defense conundrum” in plants (Chini et al., 2007; Huot et al., 2014), including in *Verticillium*-host interactions (Dhar et al., 2019). In Verticillium wilt of oilseed rape, the transition to reproductive stage is critical for the spread of the pathogen and appearance of disease symptoms, while in cotton, the severity of Verticillium wilt increases in the field is concomitant with the transition to flowering (Li et al., 2014). The cross-talk between the aspartate-glutamate-leucine-leucine-alanine (DELLA) proteins that are negative regulators of plant growth and jasmonate zim domain (JAZ) proteins that are negative regulators of plant defense response appears to be involved in these interactions. While DELLA proteins negatively regulate the GA signaling pathway involved in plant growth, JAZ proteins negatively regulate the JA signaling pathway involved in plant defense response (Hou et al., 2010). *WRKY* genes belong to a family of transcription factors that are involved in multiple processes, including growth, defense, and various hormone signaling. One such member of the family in cotton (*GbWRKY1*), isolated from a bacteriophage full-length cDNA library, is induced by *V. dahliae* as well as by the phytohormones SA, JA, and ET (Shu-Ling et al., 2012). The cotton *GbWRKY1* has also been shown to be a negative regulator of defense response against *Verticillium* and *Botrytis*, most likely through the attenuating JA signaling pathway. *GbWRKY1* is expressed upon *Verticillium* infection as well as JA application but also binds to the *JAZ1* promoter. The overexpression of *GbWRKY1* in *Arabidopsis* also led to early flowering. Steady availability of optimal resources, combined with their sessile nature, is a limiting factor in the physiological processes of plants. Thus, plants switch between the investment in growth and development, on the one hand, and defense responses to various biotic and abiotic factors on the other, resulting in the “growth versus defense trade-off” (Chini et al., 2007; Huot et al., 2014). Taken together, the work by Li et al. (2014) is indicative of growth – defense axis that is influenced by *GbWRKY1*, with development favored over the defense in an antagonistic interaction between the JA/GA pathways.

**Figure 2:**
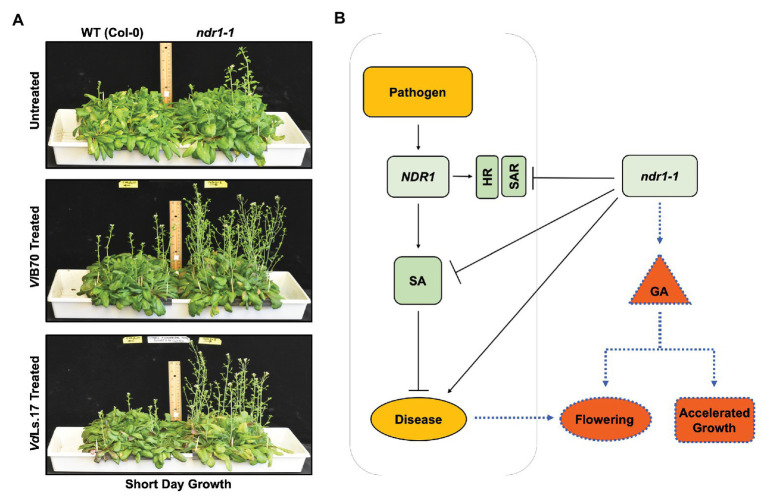
*Verticillium*-mediated acceleration in growth and flowering time in *Arabidopsis*, and the role of *NDR1* in mediating hormone cross-talk affecting the growth and defense response. **(A)** *Arabidopsis ndr1-1* mutation causes early flowering in comparison to the wild-type Col-0. *ndr1-1* plants grown under short day conditions (10/14 h light/dark cycle) flower early and bolt faster relative to the wild-type Col-0 control plants. A similar phenotype occurs in plants grown under the long day conditions (14/10 h light/dark cycle). Reproduced with permission (Dhar et al., 2019). *Arabidopsis ndr1-1* mutants are susceptible to *Verticillium longisporum* (*Vl*Bob70) and flower earlier than the corresponding uninfected plants. Plants of both genotypes were grown under short day conditions and were treated with *Vl*Bob70 at a concentration of 5 × 10^6^ conidia/ml applied directly to the soil in the 2nd week of their growth. Picture was taken at 4 weeks post infection. *Arabidopsis ndr1-1* mutants are susceptible to *Verticillium dahliae* (*Vd*Ls17) and display flowering earlier than the corresponding uninfected plants. Plants were grown, inoculated, and the picture was taken as described for *Vl*Bob70. **(B)** The *ndr1-1* mutant as a tool to dissect hormone cross-talk during pathogen infection. The *nonhost specific disease resistance 1* (*NDR1*) gene is required for defense against biotic and abiotic stresses various plant species. It has also been shown to play an important role in defense and altered growth and development response to infection by pathogenic *Verticillium* sp. While the *Arabidopsis ndr1-1* plants are defective in SA signaling rendering the plant susceptible to pathogenic *Verticillium*, on the one hand, it leads to early senescence and accelerated growth in a GA-dependent manner, on the other hand (Dhar et al., 2019), thus providing an excellent example of the “growth versus defense” response during pathogen attack. SA, salicylic acid; HR, hypersensitive response; SAR, systemic acquired resistance; GA, gibberellic acid; *ndr1-1, nonhost specific disease resistance 1-1* mutant.

The well-characterized *Arabidopsis ndr1-1* defense mutant (Century et al., 1995; Aarts et al., 1998), which confers increased susceptibility to *Verticillium* infection, was shown to display early flowering (Figure 2A top panel) linked to enhanced expression of GA biosynthesis genes, increased bioactive GA *in planta* as well as a reduction in the levels of DELLA components (Dhar et al., 2019). Because *NDR1* is central to SA-mediated defense responses, the establishment of SAR, and the HR response against pathogens (Century et al., 1995; Aarts et al., 1998), this new finding raises the exciting possibility that increased susceptibility in *ndr1-1* might not only be due to its inability to accumulate SA but also due to the fact that enhanced GA signaling in this mutant might also contribute toward this outcome (Figure 2B). Though *ndr1-1* plants were resistant to GA inhibitor paclobutrazol (PAC) at physiologically relevant concentrations, the mutant plants responded to external GA application, which further accelerated its flowering time indicative of a functionally responsive GA signaling pathway. When the *ndr1-1* mutant and wild type plants were treated simultaneously with GA and PAC, a reversion to wild type level in flowering time was observed for the *ndr1-1* mutants indicating that the GA biosynthesis, not perception was affected. At the same time, *Verticillium* infection further accelerated the bolting in *ndr1-1* plants (Figure 2A middle and bottom panel), suggesting that the pathogen infection promoted a faster transition to the reproductive stage while compromising resistance to the pathogen caused by the genetic lesion in this mutant. Furthermore, work using this GA signaling enhanced *ndr1-1* mutant appears to support a model, where inhibition of GA signaling might lead to enhanced resistance to *Verticillium*.

Mutation in *NDR1* renders *Arabidopsis* defective in the accumulation of SA and potentiation of SAR in the face of a pathogen challenge (Century et al., 1995). Thus, the reduced defense response accompanied by an early transition to flowering in a GA-dependent manner in the *ndr1-1* mutant seems to further add to the assumption that resistance to pathogen comes at the cost of normal plant growth and development (Figures 1, 2). This work points toward an important role of GA signaling during *Verticillium* wilt disease development.

### Role of Auxin in *Verticillium* Resistance

The expression levels of the auxin receptor genes *transport inhibitor response 1* (*TIR1*), *auxin signaling F box protein 1* (*AFB1*), and *auxin signaling F box protein 3* (*AFB3*) as well as that of the auxin transporter gene *auxin resistant 4* (*AXR4*) were upregulated in *A. thaliana* roots following infection by *V. dahliae*. However, the levels of indole-3-acetic acid (IAA) between the control and the pathogen-treated wild type *A. thaliana* plants were not significantly different. Interestingly, decreased symptom severity and increased resistance to the pathogen occurred in the pathogen treated *afb1*, *afb3*, and *axr4* mutant plants. The increased resistance to the pathogen in *afb1* and *axr4* was attributed to the upregulation of the defense-related *PR1* (a component of the SA pathway). In contrast, the pathogen resistance in the *afb3* mutant was attributed to an increased level of *PDF1.2* expression (a component of the JA pathway; Fousia et al., 2018). Thus, manipulation of auxin signaling and not its biosynthesis in the root system of *V. dahliae* infected plants may promote Verticillium wilt in susceptible hosts. The activation of the auxin signaling pathway in plant roots might potentially be used as a strategy by the pathogen to suppress the SA dependent host defense responses during the initial biotrophic phase of *V. dahliae* (a hemibiotroph).

In a separate study, 19 strains from 10 *Verticillium* spp. showed that volatile compounds (VC) from *Verticillium* caused preferential allocation of resources to drive root growth over shoot growth by manipulating auxin (AUX) signaling pathways. Inhibition of the AUX signaling pathway using an auxin efflux inhibitor compromised this change in growth pattern, underscoring the role of AUX during Verticillium wilt. Furthermore, several components of the AUX signaling pathway, including *TIR1*, *TIR3*, *AUX1*, and *AXR1* were involved in the regulation of VC-mediated plant growth (Li et al., 2018a).

### Role of Nitric Oxide and Other Growth-Promoting Substances in *Verticillium* Resistance

A preinoculative soil drenching with β-aminobutyric acid (BABA) induced resistance to *V. longisporum* infection of a susceptible oilseed rape (*B. napus*) cultivar and prevented plant stunting as the disease progressed (Kamble et al., 2013). An early but significant increase in the activity of PAL led to higher synthesis and accumulation of phenylpropanoids, as evidenced by an higher number of cells surrounding the xylem vessels storing phenol was proposed to underlie this chemically induced resistance. This change in vascular architecture, in turn, led to the containment of the pathogen by inhibiting colonization of the shoot (Kamble et al., 2013).

NO is a gaseous hormone that is mostly implicated in ROS and defense signaling in plants. *Verticillium* infection and *Vd* toxins induce cell death with the active involvement of ROS (Jia et al., 2007; Zhang et al., 2013), while *Vd* toxin alters hormone balances during *Verticillium* infection (Pegg and Brady, 2002). Both NO and H_2_O_2_ are produced in cotton suspension cells treated with *Vd* toxin (Jia et al., 2007). Nitric oxide synthase (NOS) and nitrate reductase (NR) enzymes have been implicated in the production of NO in plants. *Vd* toxin induces NO in the leaves of *Arabidopsis* plants with peak activity around an hour post-treatment though this induction was strongly suppressed in the NR deficient *nia1 x nia2* mutant (Shi and Li, 2008). Further, the *Vd* toxin exerted its cell death-inducing effect through alterations of cytoskeleton and nucleoli. NO production induced by *Vd* toxin caused depolymerization and destabilization of the cortical microtubules rather than the actin microfilaments inside the cell, accompanying an induced cell death resembling HR that coincides with the activation of defense response in the host cells (Shi et al., 2009). While this is an interesting example, few *Verticillium* effectors are known to actively interfere in changing the hormone levels in the host plants (Liu et al., 2014; Qin et al., 2018).

Results from studies involving VIGS-mediated silencing of a coiled-coil (CC)-NBS-LRR-type gene (*GbRVd*) from a resistant cotton cultivar rendered the cotton plants susceptible to *V. dahliae* infection by downregulation of SA, NO, and H_2_O_2_ production (Yang et al., 2016). Another study utilizing *Vd* toxins and mutants blocked nitrate reductase-mediated NO production, demonstrating that H_2_O_2_ functions upstream of NO to modulate the dynamic microtubule cytoskeleton and leading to defense activation during *V. dahliae* infection through the activity of nitrate reductase (Yao et al., 2012).

## Summary

Verticillium wilts result in alterations in the hormone signaling pathways that in turn impact plant development, reproduction, and metabolism, as well as responses to pathogen infection.

**Figure 3:**
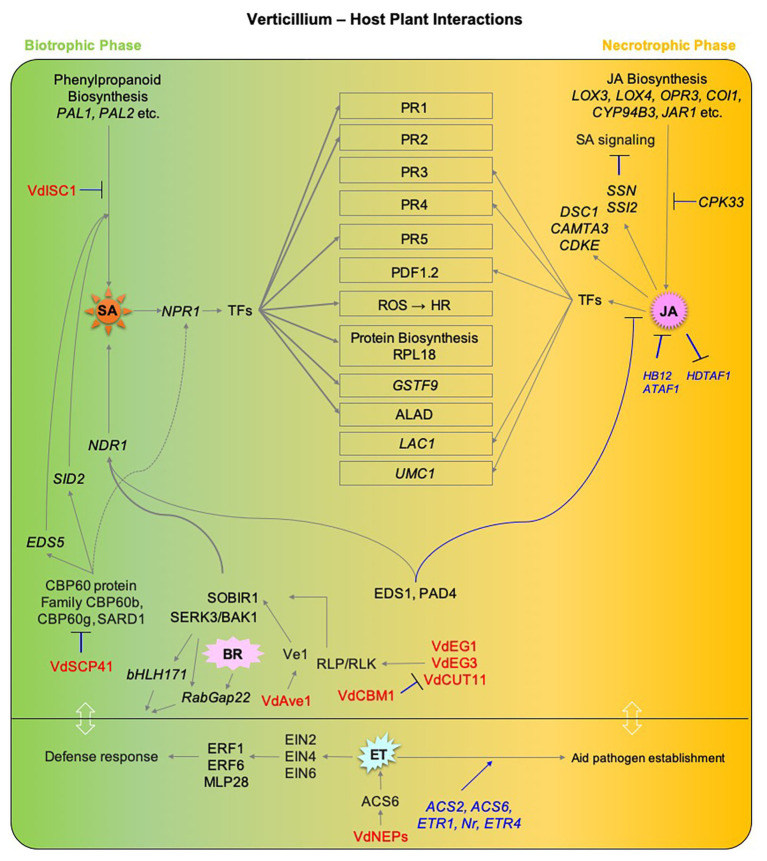
An overview of the major hormone signaling pathways involved in defense responses to *Verticillium* spp. Components of major hormone signaling pathways at play during *Verticillium* spp. infection of host plants. Major phytohormone pathways including SA, JA, ET, and BR are affected during the progression of Verticillium wilt. *Verticillium dahliae* is a hemibiotrophic pathogen that has an initial biotrophic phase (shown in green), following host colonization and death, the pathogen exhibits a necrotrophic lifestyle (shown in orange). SA responses play a major role during the biotrophic phase while JA/ET-mediated defenses are largely active later during the switch to necrotrophic phase. Gray lines with arrowheads represent positive regulation, while blue lines with a black head represent negative regulation. Host genetic components involved in negative regulations are shown in blue. *Verticillium* spp. factors that influence phytohormone pathways directly in the host are shown in red. Components involving studies on PROTEIN are in plain CAPS while those involving *GENE* are *ITALICIZED*. TFs, Transcription factors; SA, SALICYLIC ACID; *PAL1*, *PHENYLALANINE AMMONIA-LYASE 1*; *PAL2*, *PHENYLALANINE AMMONIA-LYASE 2*; *NPR1*, *NONEXPRESSER OF PR GENES 1*; *SID2, SALICYLIC ACID INDUCTION DEFICIENT 2*; *EDS1, ENHANCED DISEASE SUSCEPTIBILITY 1*; *EDS5, ENHANCED DISEASE SUSCEPTIBILITY 5*; *PAD4, PHYTOALEXIN DEFICIENT 4*; *NDR1, NON RACE-SPECIFIC DISEASE RESISTANCE 1*; *ALAD, 5-AMINOLEVULINIC ACID DEHYDRATASE*; *GSTF9, GLUTATHIONE S-TRANSFERASE PHI 9*; *SARD1, SAR DEFICIENT 1*; *CBP60b, CAM-BINDING PROTEIN 60b*; JA, JASMONIC ACID; *LOX3, LIPOXYGENASE 3*; *LOX4, LIPOXYGENASE 4*; *OPR3, 12-OXOPHYTODIENOATE REDUCTASE 3*; *OPR4, 12-OXOPHYTODIENOATE REDUCTASE 4*; *COI1, CORONATINE INSENSITIVE 1*; *CYP94B3, CYTOCHROME P450*_*FAMILY 94_SUBFAMILY B_POLYPEPTIDE 3*; *JAR1, JASMONATE RESISTANT 1*; *LAC1, LACCASE 1*; *UMC1, UMECYANIN-LIKE 1*; *CPK33, CALCIUM-DEPENDENT PROTEIN KINASE 33*; *CAMTA3, CALMODULIN-BINDING TRANSCRIPTION ACTIVATOR 3*; *DSC1, DOMINANT SUPPRESSOR OF CAMTA3 1*; CDKE, CYCLIN-DEPENDENT KINASE E; *SSN, SILENCE-INDUCED STEM NECROSIS*; *SSI2, SUPPRESSOR OF SA INSENSITIVE 2*; *HDTF1, HOMEODOMAIN TRANSCRIPTION FACTOR 1*; *HB12, HOMEOBOX 12*; *ATAF1, ARABIDOPSIS THALIANA ACTIVATING FACTOR 1*; ET, ETHYLENE; *ETR1, ETHYLENE RESISTANT 1*; *ETR4, ETHYLENE RESISTANT 4*; *Nr, NEVER RIPE*; *ACS2, 1-AMINOCYCLOPROPANE-1CARBOXYLIC ACID SYNTHASE 2*; *ACS6, 1-AMINOCYCLOPROPANE-1CARBOXYLIC ACID SYNTHASE 6*; *EIN2, ETHYLENE INSENSITIVE 2*; *EIN4, ETHYLENE INSENSITIVE 4*; *EIN6, ETHYLENE INSENSITIVE 6*; *ERF1, ETHYLENE RESPONSE FACTOR 1*; *ERF6, ETHYLENE RESPONSE FACTOR 6*; *MPL28, MAJOR LATEX PROTEIN 28*; BA, BRASSINOSTEROID; BAK1, BRI1-ASSOCIATED RECEPTOR KINASE; SOBIR1, SUPPRESSOR OF BIR1 1; *bHLH171, BHLH-TYPE TRANSCRIPTION FACTOR 171*; *RABGAP22, Rab GTPase-ACTIVATING PROTEIN 22*; *Ve1, VERTICILLIUM Race1 RESISTANCE*; *Vd, Verticillium dahliae*; VdEG1, *V. dahliae* ENDOGLUCANASE 1; VdEG3, *V. dahliae* ENDOGLUCANASE 3; VdCUT11, *V. dahliae* CUTINASE 11; VdCBM1, *V. dahlia* CARBOHYDRATE-BINDING MODULE FAMILY 1; VdAve1, *V. dahliae* Avirulence on Ve1; VdSCP41, *V. dahliae* SECRETORY PROTEIN 41; VdISC1, *V. dahliae* ISOCHORISMATASE 1; *Vd*NEP, *V. dahliae* NECROSIS-AND ETHYLENE-INDUCING PROTEIN.

Host defense responses to *Verticillium* infection involve components of hormone signaling pathways, which may act in synergistic or antagonistic ways and result in different outcomes. Furthermore, the nature of this interaction between hormone pathways depends on the organ and stage of the infection process. Nonetheless, there is an overall consensus that SA plays a major role in regulating defense responses and establishing resistance during the initial biotrophic phase of *Verticillium* spp. infection, while JA signaling is more prominent following the establishment of the pathogen in the vasculature and a switch to necrotrophic lifestyle. Thus, pathway components of either of these hormones seem to affect resistance against *Verticillium* spp. in an antagonistic manner (Figure 3). In contrast, the role of ET is somewhat intriguing during the disease cycle of *Verticillium*. While ET perception and signaling is required for normal defense response during the initial stages of the pathogen attack, it seems that *Verticillium* spp. take advantage of the role of ET in senescence and cell death-promoting processes after switching to a necrotrophic phase to utilize the dying host tissues as a source of nutrients to produce resting structures. The fact that ET signaling plays a major role in promotion and regulation of senescence and during *Verticillium* infection suggests that an elevated ET signaling may offer an advantage to the host to defend against the pathogen during early stages of infection while this advantage shifts to the pathogen when the host is compromised for the production of resting structures later. Alternatively, the pathogen may trigger ET signaling, accelerating senescence thereby completing its life cycle on senescing and decaying plant tissues (Figure 3). In this respect, establishing an active and direct line of evidence addressing the “cause or effect” conundrum by correlating the lifestyle switch in the pathogen to host developmental senescence, and the exact nature and role of ET signaling in both the pathogen and host will help advance our understanding on this aspect of Verticillium wilt progression.

Though the function of *Verticillium* effectors and their effect on plant defense have been studied, not much is known about their ability to directly alter/influence hormone signaling in the host plants. However, as summarized in Figure 3, a few studies on the pathogen’s ability to directly interfere with host hormone levels are available. Among the known *Verticillium* effectors that directly influence the host hormone signaling are some that alter the biosynthesis of SA in the host, a few that influence the host BR signaling, and a single one that influences ET signaling (Liu et al., 2014; Qin et al., 2018; Tsolakidou et al., 2020).

**Figure 4:**
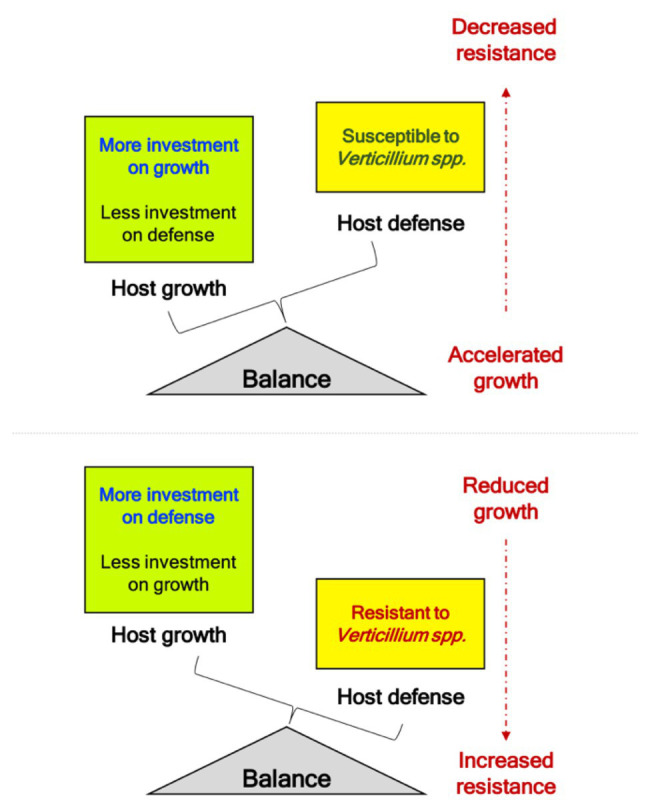
Balance of “growth and defense” during challenge to phytopathogenic *Verticillium* spp. Studies linking *Verticillium* resistance to genes like *NDR1*, *ALAD*, and *GhSSN* provide evidence that altered resistance to this soilborne pathogen results in growth defects. Mutations in gene like *ALAD* and *SSN* that result in fortified defenses comes at the cost of the plant growth (refer Summary section for details). Alternatively, mutations like *ndr1-1* that result in susceptibility to pathogenic *Verticillium* spp. result in accelerated growth, and faster transition to reproductive competent stage marked by early transition to flowering to propagate the genetic material (refer Figure 2). When the pathogen attack subdues the plant defense responses, an overall irreversible decline in plant growth results with the host succumbing to the pathogen marking the end to a successful infection (susceptibility).

Volatiles emitted by pathogenic *Verticillium* spp. promote growth in *Arabidopsis* through the AUX signaling pathways (Li et al., 2018a). Additionally, other growth-related hormones such as GA and BR are upregulated during the initial phases of the pathogen attack, suggestive of a response involves a hastening of the propagation of its genetic material by accelerating development when under threat from the pathogen. While many receptor-like kinases (RLK) involved in BR signaling were initially associated with cell growth and development, their role in defense against multiple pathogens has led to an interesting paradigm in terms of “growth versus defense” (Huot et al., 2014). Alternately, this initial spurt in host growth may be stimulated by the pathogen to and the hastening of host growth and remobilizing nutrients under a perceived threat could be subverted to support pathogen growth, colonization, and completion of disease cycle on the host. The identification of the genetic components underlining this “growth versus defense” framework will provide an interesting insight into the pathogenic *Verticillium* infection strategies since disease symptoms also seem to manifest and overlap with the onset of flowering – a developmental phase marking the transition of the plant from the vegetative to the reproductive state. While the transition to flowering is a progressive developmental phase, it also coincides with the appearance of visible developmental senescence, which in turn also seems to coincide with the manifestation of disease symptoms (Sadras et al., 2000). Thus, resistance to pathogenic *Verticillium* spp. (involving SA, JA, and ET pathways) seems to be intricately tied with host developmental transition to reproductive phase (mostly involving AUX and GA) and progression of developmental senescence (mostly involving ET and ABA). However, these three fundamental plant processes are coordinated by an intricate network of hormone signaling. Therefore, studies involving hormone relations at an organ-specific and whole plant scale during the disease cycle will help identify the genetic components and signaling networks that connect growth, defense, senescence, nutrient remobilization, and development during the progression of the Verticillium wilt. The *ndr1-1* mutation, which decreases resistance to *Verticillium* in an otherwise normal plant, leads to early senescence and accelerated flowering (Figure 2A). While lower disease resistance in *ndr1-1* has been attributed to a defect in SA signaling (Century et al., 1995), the accelerated flowering is the result of increased GA signaling, an essential component of floral transition or reproductive competency in *Arabidopsis* (Dhar et al., 2019). This inverse relationship in SA-mediated defense and growth/development promotion by GA strengthens the inverse relationship underlining the “growth versus defense” paradigm. As the *ndr1-1* mutation causes susceptibility to pathogens, rapid growth, and early transition to flowering, the plant is perhaps “primed” for pathogen attack as a result of the availability of additional metabolic resources diverted from defense (Figures 2, 4). This line of reasoning seems more likely when considering studies on mutations in genes like *ALAD* and *GhSSN* that resulted in increased resistance to Verticillium wilt. Such mutations caused hyperaccumulation of defense-related hormones SA and JA, respectively, while leading to reduced overall growth and developmental defects in the plants (depicted in Figure 4; Sun et al., 2014; Chai et al., 2017).

With successful establishment of the pathogen in a susceptible host, plant growth accelerates initially only to decline progressively as the infection manifests in such host plants. In resistant hosts, the increased tolerance to this vascular pathogen seems to come at the cost of host growth inhibition, with the diversion of resources to fortify host defense responses (Figures 2, 4). Under normal conditions, the BR pathway is a major growth-related hormone pathway that also is involved in defense responses against pathogens. Unsurprisingly current findings support that the steroid phytohormone signaling components, including *BRI1*, *BIK1*, and *SOBIR1*, are involved in regulating basal defense against other pathogens while contributing to various aspects of plant growth, light responses, and development (Yu et al., 2018).

Evidence of crosstalk between BR-JA and JA-GA is emerging in various systems, which may highlight the “growth-defense” paradigm in *Verticillium*-host defense. Future helpful studies may address the SA-JA antagonism underlining phase change of the pathogen during infection since this seems to influence the outcome of *Verticillium*-host interactions. Furthermore, the AUX-ET and AUX-BR synergistic interactions in the roots require greater clarification in response to and during the invasion of *Verticillium* spp. Defining such hormone interactions and signaling components in a time- and organ-specific manner during various host-*Verticillium* spp. interactions could help build a unified strategy to approach genetic manipulation of plant hormone signaling networks for broad-spectrum plant defense. Such a development could also set the stage to fabricate designer small molecules/chemical regulators to fortify plant defense or minimize the loss of commercial commodities in crop plants infected by pathogenic *Verticillium* spp. in conjunction with other traditional and biological disease management methods.

## Author Contributions

ND and J-YC prepared all figures. ND wrote the initial draft with significant inputs from SK and KS. SK, KS, and J-YC edited the manuscript. All authors contributed to the article and approved the submitted version.

### Conflict of Interest

The authors declare that the research was conducted in the absence of any commercial or financial relationships that could be construed as a potential conflict of interest. Mention of trade names or commercial products in this publication is solely for the purpose of providing specific information and does not imply recommendation or endorsement by the United States Department of Agriculture (USDA). USDA is an equal opportunity provider and employer.
